# Navigating the digital landscape: unraveling the interplay of challenge and hindrance components of technostress on employee voice behavior

**DOI:** 10.3389/fpsyg.2025.1434275

**Published:** 2025-03-26

**Authors:** Barnabás Buzás, Adél Csenge Simon, Orhidea Edith Kiss, Klára Faragó

**Affiliations:** ^1^Doctoral School of Psychology, Eötvös Loránd University, Budapest, Hungary; ^2^Institute of Psychology, ELTE Eötvös Loránd University, Budapest, Hungary

**Keywords:** voice behavior, technostress, challenge-hindrance stressors framework, psychological safety, intrinsic motivation

## Abstract

**Introduction:**

The increasing digitalization of office work, especially with the rise of remote work, has amplified the impact of technostress in organizations. This study examines how technostress influences employee voice behavior. Grounded in the challenge-hindrance stressor framework, we hypothesize that certain aspects of technostress may positively affect voice behavior, psychological safety, intrinsic motivation, and affective commitment. Our findings provide insights for organizations to understand these dynamics and develop managerial strategies that foster positive workplace behaviors.

**Methods:**

We conducted a cross-sectional study using an online questionnaire with office employees experienced in remote work (*N* = 361). Data were analyzed using three-step hierarchical regression models to assess the direct effects of technostress on voice behavior. Additionally, structural equation models (SEM) were used to explore indirect effects and the moderating roles of psychological safety, intrinsic motivation, and affective commitment.

**Results:**

Our findings reveal that technostress consists of challenge and hindrance components. Techno-uncertainty and, to a lesser extent, techno-overload acted as challenge stressors, positively influencing voice behavior directly or through intrinsic motivation and affective commitment. Conversely, techno-insecurity and techno-complexity emerged as hindrance stressors. Techno-insecurity negatively affected all measured variables, while techno-complexity reduced voice behavior and psychological safety. We observed a positive linear relationship between challenge stressors and voice behavior, a negative linear relationship with hindrance stressors, and a weak U-shaped relationship between techno-insecurity and promotive voice.

**Discussion:**

Our study underscores the need to analyze technostress through the challenge-hindrance stressors framework, as its components can both enhance and hinder employee motivation and voice behavior. We interpret our findings through the lens of Conservation of Resources (COR) theory, emphasizing a proactive rather than a defensive or reactive approach. Additionally, we propose managerial strategies to encourage voice behavior in technostress-prone work environments.

## Introduction

Recent global events, such as the COVID-19 pandemic, the Russian-Ukrainian war, the Israel-Palestine Crisis, and inflation, have made the organizational landscape even more unpredictable, emphasizing the significance of the BANI Environment (Gowri Kusuma and Sarma, 2023). In this ever-changing environment, adaptability is crucial for organizations. Organizational voice behavior is pivotal in facilitating adaptability, as it empowers employees to provide creative suggestions for addressing external challenges and threats (Rasheed et al., 2017; Um-e-Rubbab and Naqvi, 2020). Moreover, engaging in voice behavior has positive effects on individual coping abilities and well-being (Barron, 2022; Liu et al., 2021; Yousaf et al., 2019).

Voice behavior has been found to be advantageous in various ways, such as showcasing individual contributions, improving team processes, preventing potential crises, and enhancing organizational performance (Hilverda et al., 2018). There is consensus among many authors regarding the growing significance of voice, particularly amid the shift to remote work, as it fosters organizational adaptability and well-being (Kim and Lim, 2020; Prouska et al., 2022; Ta’Amnha et al., 2021). Communication is the means through which voices are articulated, nevertheless, the remote work environment and more broadly, internet-mediated communication presents a hurdle to the dialogue between leaders and employees in the workplace. Participating in digital communication presents a challenge by limiting the content and the amount of messages and the ability to convey nonverbal and metacommunicative signals (Mao and DeAndrea, 2019). Furthermore, the diminished feedback from leaders to employees’ voices amplifie the risk associated with using one’s voice and potentially reduces employees’ inclination to voice their concerns and ideas. The aim of the present study is to investigate how individuals employ their voice in response to workplace technostress. Understanding the extent to which stress accompanying the use of digital tools hinders voice behavior, as well as the mechanisms through which it does so is crucial. Therefore, we aim to explore how the ongoing adaptation to a constantly evolving digital toolset and the resulting technostress influence organizational voice behavior.

Remote work has gained prominence in recent years, especially during the pandemic, with some experts even considering it the new norm (Davis et al., 2020). The adoption of digital tools and practices has been instrumental in making remote work feasible (Leonardi, 2021). However, this extensive digitalization has brought about the phenomenon of technostress, (“any negative impact on attitudes, thoughts, behaviors or psychology caused directly or indirectly by technology,” Weil and Rosen, 1997, p. 5) which is the central focus of this study regarding its impact on voice behavior and, consequently, organizational adaptability and innovativeness.

Job stressors are linked to negative work-related outcomes and are known to significantly inhibit voice behavior as well. While existing literature has delved into the connections between job stressors and voice behavior (Ng and Feldman, 2012; Song et al., 2017; Xia et al., 2020; Yan and Xiao, 2016; Zhou et al., 2019) further research is still needed in this area. Previous research conducted by our team has shown that crises, remote work, and isolation do not necessarily reduce the frequency of voice behavior when there is a high level of leadership openness to opinions (Buzás and Faragó, 2023). Given that a significant portion of employees express a desire to continue remote work practices that became prevalent during the COVID-19 pandemic (Simon et al., 2023), we propose that technostress may not exclusively have a negative impact on voice behavior.

To understand the possible positive effects of technostress, we refer to researchers’ suggestion to categorize job stressors into challenge stressors, which offer opportunities for personal growth and rewards, and hindrance stressors, which hinder personal growth and goal attainment (Abbas and Raja, 2019). Challenge stressors are associated with positive work attitudes and voice behavior, as they encourage employees to increase their commitment with the expectation of receiving rewards after overcoming the stressors. On the other hand, the relationship between hindrance stressors and voice behavior is not well understood (Zhou et al., 2019). Some perspectives suggest that hindrance stressors discourage employees from engaging in extra-role behavior and are negatively related to voice behavior (Yan and Xiao, 2016). Conversely, there’s also an argument that hindrance stressors might be positively related to voice behavior, as expressing concerns about detrimental behavior could help in removing hindrance stressors (Song et al., 2017; Xia et al., 2020; Zhou et al., 2019). The dynamic effect of challenge and hindrance stressors on job outcomes is explained through the Conservation of Resources Theory (Hobfoll, 1989, 2002). When employees experience stress, they may not have the resources (time, energy) to engage in voice behavior. However, engaging in voice behavior can help employees acquire additional resources and reduce stress. Exploring how people react to disadvantageous circumstances provides an opportunity to assist employees in adapting.

Only a limited number of studies have explored technostress within the challenge-hindrance stressors framework (Chandra et al., 2019), and there has been relatively little research focusing on the relationship between voice behavior and challenge-hindrance stress (Hamrick et al., 2024; Ng and Feldman, 2012; Song et al., 2017; Tangirala et al., 2013; Xia et al., 2020; Zhou et al., 2019). For example, in the context of remote work, Hamrick et al. (2024) found that interactional remote monitoring is appraised as a challenge and encourages voice, while observational monitoring is appraised as a hindrance and fosters silence.

### Contribution to the existing knowledge

Our research addresses an important gap in the literature by exploring the challenging or hindering effect of technostress on voice behavior. To the best of our knowledge, this is the first study to investigate the relationship between voice behavior and technostress, focusing on the differentiation of separate technostress factors. Our study makes three primary contributions.

First, we aim to elucidate the theoretical foundation and provide empirical evidence for the connection between technostress and voice behavior. By doing so, we seek to uncover the underlying dynamics and mechanisms that drive employee motivation in stressful situations.

Second, we classify the five factors of technostress as either challenge or hindrance stressors and examine the relationships between each technostress creator and employee voice behavior. By making this distinction, we aim to offer insights into which aspects of technostress motivate or hinder voice behavior. This nuanced approach moves beyond treating technostress as a single construct and enables us to provide managers with targeted recommendations based on the specific technostress factors most influential in either promoting or inhibiting voice behavior.

Finally, expanding upon the existing body of literature, we explore the moderating effects of psychological safety, intrinsic work motivation, and affective commitment to the relationship between stressors and performance. All three constructs have been extensively documented as predictors of voice behavior (Detert and Burris, 2007; Edmondson and Lei, 2014; Galletta et al., 2011; Jaaffar and Samy, 2023; Kuvaas, 2006; Liang et al., 2012; Uğurlu and Ayas, 2016) and it can be argued for each of them that they are particularly sensitive to increases in workplace stress levels (Bočková and Lajčin, 2021; Chua Wan Lin, 2020; Hertel et al., 2005; Kohont and Ignjatović, 2022; Lechner and Tobias Mortlock, 2022; Schade et al., 2021; Wang et al., 2020). These factors serve as supplementary resources that help employees better cope with work-related stressors, thereby enhancing their effectiveness (Luo, 1999; Tetteh et al., 2020).

Our results can assist organizations to understand better how technostress factors influence employee voice behavior. This knowledge can support the development of managerial strategies to foster positive activities within organizations, offering practical implications for managers to optimize employee voice in the context of digital work and technostress. Additionally, our findings highlight the importance of considering individual perceptions of technostress and underscore the potential benefits of personalized mentoring in addressing these challenges.

## Literature review and hypothesis development

### Voice behavior

The importance of voice behavior has been increasingly recognized, particularly with the expansion of work-from-home (WFH) opportunities. Employee voice plays a pivotal role in enhancing an organization’s adaptability, innovativeness, and overall well-being (Kim and Lim, 2020; Prouska et al., 2022; Ta’Amnha et al., 2021). However, there are significant barriers to speaking up within the workplace, including concerns about leader or coworker dissent (Ng and Feldman, 2012) and challenges related to digital communication (Mao and DeAndrea, 2019). Overcoming these obstacles and promoting a culture of open communication and voice behavior is crucial for organizations aiming to thrive in an ever-changing work environment.

Organizational voice behavior refers to the intentional expression of employees’ suggestions and criticisms aimed at influencing organizational activities to improve productivity and development (Bowen and Blackmon, 2003; Van Dyne and LePine, 1998). Liang et al. (2012) identified two dimensions of organizational voice behavior: promotive voice, which involves sharing ideas and offering constructive suggestions to enhance efficiency and effectiveness within the organization, and prohibitive voice, which entails expressing concerns regarding potential threats, malicious organizational behavior, and poor performance. Promotive voice looks forward and focuses on how the organization can operate more efficiently while prohibitive voice focuses on the present and past, highlighting existing flaws and issues. Both promotive and prohibitive voice behaviors share common characteristics: they are voluntary actions taken by employees, and they are consistently driven by a desire to be helpful and improve the organization. Nonetheless, leaders frequently perceive suggestions as a threat to their position (Miceli et al., 2008; Morrison, 2014), while employees harbor concerns about potential negative outcomes, including negative performance evaluations, being assigned undesirable tasks, or facing disapproval from colleagues (Morrison, 2011).

Chamberlin et al. (2017), along with Morrison (2023), emphasize that most studies still do not clearly distinguish between promotive and prohibitive voice, highlighting the need for further clarification. While some earlier studies (Lin and Johnson, 2015) have reported similar findings for both types of voice, others (Kakkar et al., 2016; Qin et al., 2014; Zhu and Wu, 2023) have identified notable differences. Wei et al. (2015) suggest that cultural values, particularly power distance, may influence the two forms of voice differently.

Employees often consider two critical factors before engaging in voice behavior, perceived efficacy, and perceived safety. Employees may hesitate to voice their opinions if they believe their voices will go unheard by leaders. Therefore, the perception of leadership openness - where leaders are receptive to employee input - is a crucial predictor of voice behavior (Ashford et al., 1998; Buzás and Faragó, 2023). Employees are more likely to engage in voice behavior when they feel safe and comfortable expressing their opinions and criticisms. A supportive atmosphere where colleagues and team leaders are receptive to feedback fosters this sense of safety (Liang et al., 2012).

Voice behavior is considered as one type of Organizational Citizenship Behavior (OCB) (Liang et al., 2012). OCB is defined as “individual behavior that is discretionary, not directly or explicitly recognized by the formal reward system, and that in the aggregate promotes the effective functioning of the organization” (Organ, 1988, p. 4.). Although OCB does not come with formal rewards, those who engage in it are generally perceived as higher performers by their leaders, which can lead to positive individual outcomes in the long run (Spitzmuller et al., 2008). However, voice behavior is a special form of citizenship behavior that also carries risks (Liang et al., 2012).

### Technostress

Technostress is indeed a growing concern in the field of organizational psychology, especially in the context of remote work (Farmania et al., 2022). It is characterized by the negative effects of technology on various aspects of individuals’ attitudes, thoughts, behavior, and even physiological responses (Weil and Rosen, 1997). The Technostress Creators Model (Berg-Beckhoff et al., 2017; Dragano and Lunau, 2020; La Torre et al., 2019) provides a framework for understanding the five central components of technostress (Tarafdar et al., 2007): (1.) Techno-overload: This refers to the overwhelming demands of work involving digital technologies, such as constant multitasking, frequent interruptions, longer working hours and pressure to respond quickly to digital communication. (2.) Techno-invasion: It involves the blurring of boundaries between work and personal life due to the flexibility offered by digital devices. Employees may find it challenging to disconnect from work. (3.) Techno-complexity: This component relates to the perception that new digital technologies are complex and difficult to master, creating challenges for employees. (4.) Techno-insecurity: Employees may fear job loss or a decline in status due to the assumption that either new digital technologies or more skilled individuals using them might replace them. (5.) Techno-uncertainty: This aspect represents the uncertainty caused by ongoing digital transformation processes or constant changes in technology (Dragano and Lunau, 2020; Ragu-Nathan et al., 2008; Torres, 2021).

The negative effects of technostress on individuals and organizations have been well-documented (La Torre et al., 2019; Salazar-Concha et al., 2021; Tarafdar et al., 2007, 2011). It can lead to reduced life satisfaction, job satisfaction, productivity, and work-life balance. It also contributes to physical and emotional strain, burnout, anxiety, and depression. On the organizational side, technostress can diminish organizational commitment (Ungku Norulkamar et al., 2009), trust, and innovativeness, while increasing role ambiguity (La Torre et al., 2019; Salazar-Concha et al., 2021; Tarafdar et al., 2007, 2011).

While existing literature predominantly focuses on negative psychological responses, further exploration of the eustress subprocess is needed. Xia (2023) emphasizes the need for future research to take a balanced approach in examining both the positive and negative aspects of technostress in human-AI collaboration. A few empirical studies have made a step in this direction, suggesting that technostress may have certain positive effects (Chandra et al., 2019; Rath-Cullimore, 2019; Schoch, 2023; Shi et al., 2023). Understanding the nuances of technostress and its implications is crucial for organizations, especially as technology continues to play a vital role in the modern workplace.

### Theoretical background of the relationship between stress and voice behavior

Most of the studies investigating the relationship between workplace stress and voice behavior are based on the Conservation of Resources Theory (Hobfoll, 1989, 2002). Ng and Feldman (2012) suggested in their meta-analysis that COR is a relevant model to examine the relationship between voice and workplace stress. This theory suggests that individuals strive to acquire and protect valuable resources. Individuals experiencing stress may refrain from voicing their opinions to conserve resources, given the risks and the time and energy required to suggest changes (Bolino and Turnley, 2005; Detert and Burris, 2007; Organ, 1988). Conversely, voicing concerns can serve as a means to protect existing resources by changing unfavorable circumstances and a strategy to acquire additional resources for the future (Dundon and Gollan, 2007). COR theory emphasizes a proactive rather than a defensive or reactive approach.

Zhou et al. (2019) also based their study on COR theory but took it a step further by incorporating the challenge-hindrance stressors framework (Cavanaugh et al., 2000). While workplace stressors generally generate strain and anxiety, challenge and hindrance stressors elicit different behavioral reactions (Podsakoff et al., 2007). COR theory suggests that when employees encounter challenge stressors, they are motivated to increase their efforts to overcome the strain. Challenge stressors are seen as opportunities for personal growth and resource acquisition, while hindrance stressors are perceived as threats that lead to resource conservation and decreased performance. However, voice behavior can mitigate hindrance stressors by promoting effective solutions or identifying ineffective or harmful behavior. Zhou et al. (2019) suggest that while hindrance stressors may trigger a resource conservation mechanism and reduce risky voice behavior they may also activate resource acquisition motivation, encouraging employees to speak up to address obstructive factors. Their findings indicate that challenge stressors have a positive linear relationship with both promotive and prohibitive voice behaviors. In contrast, hindrance stressors exhibit a U-shaped relationship with these two forms of voice behavior—initially, as stress increases, individuals reduce voice behavior due to resource conservation concerns, but beyond a certain threshold, they increase voice behavior, adopting a resource acquisition perspective.

Zhou et al. (2019) identified work-relevant challenge and hindrance stressors. Challenge stressors include perceptions of workload, time pressure, job complexity, and responsibility as they have the potential to foster mastery, personal growth, or future benefits. Hindrance stressors encompass role conflict, role ambiguity, politics, and job insecurities they serve as limitations or obstacles to achieving goals or personal growth (Cavanaugh et al., 2000; Zhou et al., 2019). Huang et al. (2024) studied the influence of challenge and hindrance time pressure on promotive and prohibitive voice and found that challenge time pressure positively influenced promotive voice while hindrance time pressure motivated prohibitive voice.

### The direct effect of technostress on voice behavior

When designing our research, we found no existing literature that directly addressed the relationship between employee voice and technostress. However, we identified relevant findings on the relationship between innovativeness and technostress, which heavily influenced our research planning (Chandra et al., 2019). Chandra et al. (2019) noted that while technostress creators are commonly described as multidimensional constructs, researchers had not separately examined the effect of each dimension. Using Control Theory (Karasek, 1979) and COR theory (Hobfoll, 1989, 2002), they explored both linear and curvilinear relationships between technostress factors and innovative behavior. Their findings revealed a complex relationship between the distinct factors of technostress and innovative behavior. Specifically, their results suggest that different dimensions of technostress can influence non-efficiency-based outcomes in distinct ways. Some dimensions—such as techno-uncertainty and, to a lesser extent, techno-overload—exhibited a positive or non-negative linear association with innovation, acting as challenge stressors. In contrast, techno-invasion and techno-complexity showed a linear negative association with innovation, categorizing them as hindrance stressors. Additionally, techno-overload, techno-invasion, and techno-complexity displayed U-shaped relationships with innovation. Notably, techno-insecurity could not be classified clearly as either a challenge or hindrance stressor.

To formulate our hypothesis, we pre-categorize the five dimensions of technostress into challenge and hindrance stressors. Our classification approach is methodically grounded in a thorough examination of the technostress literature and the items in the Technostress Creators Inventory (Ragu-Nathan et al., 2008; Tarafdar et al., 2007). We classify techno-overload as a challenge stressor, as it is described as such in the literature. The corresponding items primarily focus on increased work speed, intensity, and time management, suggesting opportunities for employees to improve efficiency. Techno-uncertainty is also considered as a challenge stressor. Although it involves constant technological change, the items do not explicitly mention stressful effects. Instead, employees may perceive this dimension as an opportunity to develop new skills and practice. Conversely, techno-insecurity is classified as a hindrance stressor as the items express a fear of job loss or an inability to cope with technological challenges, posing a threat to job security and control. Techno-complexity is also categorized as a hindrance stressor because it represents difficulties in mastering complex technologies, which can be perceived as obstacles to achieving task efficiency and performance. Finally, techno-invasion is considered a hindrance stressor because it involves blurred boundaries between work and personal life, creating obstacles to personal well-being and work-life balance (Ragu-Nathan et al., 2008; Tarafdar et al., 2007).

As we noted earlier, there is a need to further explore the distinct aspects of promotive and prohibitive voice. Given the ambiguity in the literature on this matter, we do not propose a specific hypothesis regarding their differences. Instead, we examine separately the relationships between promotive and prohibitive voice and technostress factors to provide further clarification.

We are formulating our first two hypotheses based on the research conducted by Zhou et al. (2019) and Chandra et al. (2019). Both studies identified a positive linear relationship between challenge stressors and the behavioral variables they investigated. We anticipate that challenge stressors (techno-overload and techno-uncertainty) will have a positive impact on voice behavior because they motivate employees to seek opportunities for improvement. Techno-overload and techno-uncertainty can be interpreted as challenge stressors, therefore…

- H1: Techno-overload and techno-uncertainty, as challenge stressors have a direct positive relationship with both promotive-and prohibitive voice (Zhou et al., 2019).

In contrast, we expect hindrance stressors (techno invasion, techno-complexity, and techno-insecurity) to have a negative effect on voice behavior, as they may hinder employees due to perceived threats or obstacles.

- H2: Techno-invasion, techno-complexity, and techno-insecurity as hindrance stressors have a direct negative effect on promotive-and prohibitive voice behavior (Chandra et al., 2019; Ng and Feldman, 2012; Xia et al., 2020).

Zhou et al. (2019) posits that both promotive and prohibitive voice behaviors are strongly associated with hindrance stressors, too. As hindrance stressors accumulate but remain below a certain threshold (referred to as the inflection point), employees tend to suppress their voice behavior due to a motivation to conserve resources. However, when hindrance stressors surpass the inflection point, employees are inclined to voice their concerns (prohibitive voice). Simultaneously, they may also propose new ideas to counteract resource losses (promotive voice). Therefore, we anticipate a curvilinear (U-shaped) relationship where employees are more likely to engage in voice behavior when faced with either high or low levels of hindrance stressors, but less likely to do so at moderate levels (Zhou et al., 2019; Chandra et al., 2019; Xia et al., 2020).

- H3: Techno-complexity, techno-insecurity, and techno-invasion have a U-shaped relationship with promotive-and prohibitive voice behavior (Zhou et al., 2019, Chandra et al., 2019; Xia et al., 2020).

### Indirect effects of technostress on voice behavior

While we assume that technostress is an important antecedent of voice, we expect its direct effect to be weak (Chandra et al., 2019). Thus, we deem it crucial to incorporate variables in the study that are established antecedents of voice behavior and have the potential to moderate the influence of technostress. These are psychological safety, intrinsic motivation, and affective commitment. Firstly, extensive research has established all three constructs as key predictors of voice behavior (Detert and Burris, 2007; Edmondson and Lei, 2014; Galletta et al., 2011; Jaaffar and Samy, 2023; Kuvaas, 2006; Liang et al., 2012; Uğurlu and Ayas, 2016). Secondly, psychological safety, intrinsic motivation, and affective commitment are especially susceptible to fluctuations in workplace stress levels (Bočková and Lajčin, 2021; Chua Wan Lin, 2020; Hertel et al., 2005; Kohont and Ignjatović, 2022; Lechner and Tobias Mortlock, 2022; Schade et al., 2021; Wang et al., 2020). The incorporation of these variables allows for a comprehensive understanding of the mechanisms through which technostress influences employee voice behavior, providing valuable insights into both theory and practice.

Psychological safety is a crucial factor in organizational settings referring to an employee’s perception of how safe it is to take risks, voice concerns, or express opinions without fear of punishment or misunderstanding from colleagues (Detert and Burris, 2007; Liang et al., 2012). When psychological safety is high within a team or organization, employees are more likely to engage in voice behavior, as they feel comfortable sharing their thoughts and ideas (Edmondson and Lei, 2014; Lee and Dahinten, 2021; Liang et al., 2012; O’Donovan and McAuliffe, 2020; Walumbwa and Schaubroeck, 2009). However, challenges in online communication and technostress can reduce psychological safety, hindering employees from expressing their voices (Chua Wan Lin, 2020; Lechner and Tobias Mortlock, 2022). Technostress arises from the perception that information technology is unreliable, insecure, and uncertain, ultimately decreasing employees’ belief that it is safe to take risks (Dumont, 2020).

Intrinsic work motivation is another significant predictor of employee voice behavior (Buzás and Faragó, 2023; Jaaffar and Samy, 2023; Uğurlu and Ayas, 2016). Employees who find their work meaningful and fulfilling are more likely to express their ideas and concerns, contributing to organizational improvement (Chen et al., 2018; Hao et al., 2021). During remote work or work-from-home arrangements, the primary challenge to motivation often stems from the lack of face-to-face interactions with colleagues (Bočková and Lajčin, 2021; Buzás and Faragó, 2023; Hertel et al., 2005; Schade et al., 2021). However, some research suggests that remote work can lead to increased autonomy, which, in turn, can heighten intrinsic motivation (Kohont and Ignjatović, 2022; Schade et al., 2021).

Affective organizational commitment represents an individual’s emotional attachment, engagement, and identification with their organization (Galletta et al., 2011; Kuvaas, 2006), is linked to intrinsic work motivation, and serves as a predictor of voice behavior (Cheng et al., 2022; Lapointe and Vandenberghe, 2018; Nisar et al., 2020; Zhou et al., 2021). Additionally, it can influence voice behavior indirectly through psychological safety (Buzás and Faragó, 2023). It’s important to note that remote work, such as working from home, can sometimes lead to a decrease in affective organizational commitment due to feelings of psychological isolation (Simon et al., 2023; Wang et al., 2020). Therefore, organizations should be mindful of maintaining a sense of connection and engagement among remote employees to encourage affective commitment and, subsequently, voice behavior.

- H4/a: Techno-overload and techno-uncertainty as challenge stressors have a beneficial effect on intrinsic motivation and affective commitment, but a negative impact on psychological safety because, like other stressors, they cause strain (Podsakoff et al., 2007).
- H4/b: Intrinsic motivation, and affective commitment, by providing additional resources, moderate the relationship between techno-overload, techno-uncertainty, and voice behavior (Kim and Beehr, 2018).
- H5/a: Techno-invasion, techno-complexity, and techno-insecurity as hindrance stressors have a negative impact on psychological safety, intrinsic motivation, and affective commitment (Chua Wan Lin, 2020; Kim and Beehr, 2018; Simon et al., 2023).
- H5/b: Psychological safety, intrinsic motivation, and affective commitment moderate the relationship between techno-invasion, techno-complexity, and techno-insecurity and promotive-and prohibitive voice behavior (Podsakoff et al., 2007).

## Materials and methods

### Participants

We used an online questionnaire for data collection. The data collection took place between March and April 2022. The participants were Hungarian employees. We used the snowball sampling method, with university students supporting the data collection as recruiters. The participants of the study had to meet the following conditions: (1) they had been working for at least 2 years, (2) they spent at least a part of their work time in home office between March 2020 (the first wave of COVID-19 in Hungary) and April 2022 and (3) they worked in an office. By defining the office as a workplace, we aimed to ensure that only employees who can engage in digital work were included in our sample for investigation. To reduce social desirability bias, we explicitly stated at the beginning of the questionnaire that all data would be collected anonymously. Furthermore, for all measurement tools, we used Likert scale responses to allow participants to nuance their answers. In total 485 participants took part in the research and after data cleaning, the data of 361 participants were analyzed (231 female, 126 male). We removed cases with any missing data. The average age of our sample was 39.0 years (*SD* = 11.4 years). Eighty-eight participants were single, 93 were in a marital or cohabiting relationship without children, 163 were in a marital or cohabiting relationship with children, and 17 were single parents. Regarding their educational background, most of the sample had a higher education degree (72%, 260 individuals). Hundred and seventy-five participants worked in the private sector and 126 in the public sector. 90% (326) worked as employees, while the others were engaged in different forms of employment (e.g., as cooperative members; self-employed individuals (12) had already been excluded earlier). Examining the sample according to the organizational hierarchy, 239 subordinates, 51 middle managers, 19 top executives, and 17 firm owners were among the participants. The average percentage of work time spent in home office was 31.4% (*SD* = 34.1%). Concerning organization size and economic sector, the research sample indicated heterogeneous distribution.

### Measures

All measures not available in Hungarian were translated according to the translation guidelines by Beaton et al. (2000).

We measured technostress by the Technostress Creators Inventory (Ragu-Nathan et al., 2008; Tarafdar et al., 2007). Since its development, it has been validated in multiple languages (Dragano and Lunau, 2020; Kotek and Vranjes, 2022; Torres, 2021), but an official Hungarian translation has not been available until now. The questionnaire consists of 23 items and is divided into five factors. Participants rate the items on a 5-point Likert scale (1 = strongly disagree, 5 = strongly agree). The techno-overload factor (5 items, Cronbach’s *α* = 0.822) measures the perception of increased workload and the need to work faster due to digital work (e.g.: “I am forced to change my work habits to adapt to new technologies.”) The techno-invasion factor (4 items, Cronbach’s *α* = 0.836) captures the intrusion of work into other areas of life due to constant accessibility through digital technologies (e.g.: “I spend less time with my family due to this technology.”). The techno-complexity factor (5 items, Cronbach’s *α* = 0.831) examines the difficulty employees face in mastering the necessary digital work techniques. (e.g.: “I need a long time to understand and use new technologies.”). The techno-insecurity factor (5 items, Cronbach’s *α* = 0.805) measures employees’ fear of losing their position or status due to insufficient knowledge of digital technology tools (e.g.: “I am threatened by co-workers with newer technology skills.”). The techno-uncertainty factor (4 items, Cronbach’s *α* = 0.840) indicates the continuous digital transformation perceived within the organization (e.g.: “There are always new developments in the technologies we use in our organization.”).

We used the scale of Liang et al. (2012) to assess employee voice behavior. This scale consists of two subscales: promotive voice (e.g.: “Raise suggestions to improve the unit’s working procedure.”) and prohibitive voice (e.g.: “Advise other colleagues against undesirable behaviors that would hamper job performance.”), each comprising five items. To reduce the scale’s length, we removed one item from each subscale, based on the lowest factor loadings reported in Liang et al.'s (2012) study. The questionnaire was part of a larger study including other measures too; therefore, we had to shorten this scale. Participants rated both subscales on a five-point Likert scale in the questionnaire. We obtained satisfactory internal consistency for both subscales (Promotive voice Cronbach’s *α* = 0.90, Prohibitive voice Cronbach’s *α* = 0.88).

To assess psychological safety, we employed the items from Liang et al.'s (2012) scale. They combined items from two previous scales (Brown and Leigh, 1996; May et al., 2004) to create a five-item scale. However, we made some modifications to the scale. Firstly, we added a novel item because we felt that the original scale lacked an assessment of emotional expression (“In my workplace, I can express my true feelings”). Secondly, we rephrased one item to be reverse-coded. The original item was “In my work unit, I can freely express my thoughts,” and we modified it to “In my work unit, I cannot express my thoughts freely.” Participants rated psychological safety on a five-point Likert scale in the questionnaire. The scale demonstrated satisfactory internal consistency (Cronbach’s *α* = 0.73).

We used the Multidimensional Work Motivation Scale (Gagné et al., 2015) to assess motivation. The original scale was translated into Hungarian by Kardos et al. (2020). The scale consists of 19 items, encompassing six factors: amotivation, material external regulation, social external regulation, introjected regulation, identified regulation, and intrinsic motivation. Each factor includes three items, except for introjected regulation, which comprises four items. We measured work motivation on a seven-point Likert scale. In our model, we specifically utilized items related to intrinsic motivation (e.g.: “Why do you or would you put efforts into your current job? Because the work I do is interesting.;” Cronbach’s α = 0.92).

We measured organizational commitment using the Three-Component Model Employee Commitment Scale (Meyer and Allen, 1991, 2004), which is a widely recognized instrument. The original survey was translated into Hungarian by Kiss et al. (2012). The scale consists of three factors: affective commitment, continuance commitment, and normative commitment. Each factor was assessed with four items on a six-point Likert scale. In our model, we used the items of affective commitment (e.g.: “I would be very happy to spend the rest of my career with this organization” Cronbach’s *α* = 0.93).

### Statistical analysis

Data was first exported to SPSS 28.0 and jamovi 2.5 for preliminary analysis (e.g., demographics, means, standard deviation, correlations, and estimation of internal consistency). Subsequently, measurement models were constructed to assess the discriminant and convergent validity of the scales. Convergent validity aims to examine whether the items effectively measure the same underlying concept. Convergent validity is determined by calculating the average variance extracted (AVE) and composite reliability (CR). Acceptable values for these indicators are AVE > 0.5 and CR > 0.70, as recommended by Hair et al. (2017). Discriminant validity was evaluated using the Forner-Larcker criterion, which involves examining the square root of AVE. As all data were gathered through the same survey, the presence of common method bias was a concern. To address this issue, we adopted the common latent variable approach proposed by Podsakoff et al. (2003).

Concerning direct relationships, we examined the linear and curvilinear relationships between the technostress creator factors and promotive-and prohibitive voice behavior. Initially, we created the squared term for each factor of technostress by averaging the items of the factors and then squaring them. Furthermore, we conducted an assessment for multicollinearity and computed the variance inflation factor (VIF), revealing no notable issues related to multicollinearity (Sheather, 2009). We applied a three-step hierarchical regression model to examine the linear and curvilinear effects of individual technostress factors on voice behavior. In the first step, we introduced the control variables, then in the second step, we introduced the technostress factors individually to examine the linear relationships, and finally in the third step we introduced the squared terms of technostress factors individually to examine the curvilinear relationships. Age and gender can be significant predictive factors of employee voice (Morrison, 2023); therefore, we considered it important to control them.

To test the moderator effect of psychological safety, intrinsic motivation, and affective commitment, we developed five structural equation models (SEM) using Amos 24.0. We calculated *p*-values, confidence intervals, and *β*-values. Given the potential non-normality of the data, we opted for the robust maximum likelihood estimator. Following the recommendations of Preacher and Hayes (2008), we utilized 5,000 bootstrap replication samples. The application of bootstrapping methods is recommended when the sample size is small to moderate (Shrout and Bolger, 2002). To evaluate the model, we examined three goodness-of-fit indices along with their respective good or acceptable cut-off values (Hu and Bentler, 1999): the Comparative Fit Index (CFI; ≥0.95 good, ≥0.90 acceptable), the Tucker-Lewis index (TLI; ≥0.95 good, ≥0.90 acceptable), and the root-mean-square error of approximation (RMSEA; ≤0.06 good, ≤0.08 acceptable).

Moderation analyses are particularly important, as they allow us to infer the mechanisms underlying main effects. We worked with measured moderators because we believe that ecological validity cannot be fully ensured in the studied topic, making it impossible to collect reliable data through experimental design. However questions arise regarding the reliability of mediation and moderation analyses when the intervening variable is only measured and not manipulated (Bullock et al., 2010; Bullock and Green, 2021). The presence of moderation was based on the statistical significance of coefficients derived from equations. We used implicit procedures that involve a conventional model that deduces the moderation effect via a singular inferential examination of path relationships between independent and dependent variables (Baron and Kenny, 1986; Rasoolimanesh et al., 2021). This topic will be further elaborated on in the limitations of the study.

## Results

Descriptive data and correlations are presented in Table 1. The results for validity and reliability based on confirmatory factor analysis (CFA) are displayed in Table 2. We calculated the composite reliability (CR), average variance extracted (AVE), and factor loadings for each investigated factor. The levels of technostress, psychological safety, affective commitment, intrinsic motivation, promotive voice, and prohibitive voice were measured by summing the average scores. Furthermore, we created parcels from the items of the Technostress Creators Inventory, which indicated the latent constructs of technostress (Chen and Weng, 2019; Cole et al., 2016; Little et al., 2013; Rhemtulla, 2016; Yang et al., 2010). The use of parcels can reduce problems caused by non-normal distribution. In Table 2, you can observe that all item loadings for the constructs fall within an acceptable range. Although the average variance extracted for psychological safety did not reach 0.5, according to Fornell and Larcker (1981), it can be deemed acceptable if the composite reliability is above 0.7. To assess multicollinearity, we examined the variance inflation factors (VIF) and tolerance. A VIF value exceeding 5.0 or a tolerance below 0.2 indicates the presence of multicollinearity (Sheather, 2009). There were a few indicators where VIF exceeded 5.0, but according to Hair et al. (1995), VIF under 10.0 can be acceptable. Moreover, O’brien (2007) suggests that the removal or transformation of these variables could cause more problems, than multicollinearity itself. After applying the common latent variable approach, it was determined that 22% of the variance could be attributed to the method factor. This finding indicates that common method variance did not pose a concern in our study, allowing us to proceed with the analysis of our hypothesized models.

**Table 1:** Descriptive statistics, validity indicators, and correlations for the hypothetic model.

Scales	*M*	*SD*	Range	*α*	1	2	3	4	5	6	7	8	9
1. Technostress Overload	2.54	0.78	1–5	0.82	-								
2. Technostress Invasion	2.28	0.82	1–5	0.84	0.51***	-							
3. Technostress Complexity	2.13	0.71	1–5	0.83	0.39***	0.40***	-						
4. Technostress Insecurity	1.94	0.64	1–5	0.81	0.35***	0.39***	0.51***	-					
5. Technostress Uncertainty	2.99	0.83	1–5	0.84	0.12**	0.17**	−0.01	0.17**	-				
6. Intrinsic Motivation	4.57	1.36	1–7	0.92	0.10*	0.10*	−0.04	−0.15**	0.12**	-			
7. Affective Commitment	4.03	1.25	1–6	0.93	0.03	0.07	0.03	−0.13**	0.06	0.51***	-		
8. Psychological Safety	3.32	0.62	1–5	0.73	−0.23***	−0.22***	−0.22***	−0.35***	0.07	0.27***	0.30***	-	
9. Promotive Voice	3.34	0.77	1–5	0.90	0.06	0.03	−0.12**	−0.18***	0.11**	0.33***	0.27***	0.30***	-
10. Prohibitive Voice	2.97	0.81	1–5	0.88	0.10*	0.04	−0.04	−0.04	0.13**	0.25***	0.20***	0.28***	0.66***

M, mean; SD, standard deviation; α, Cronbach’s alpha; ***p < 0.001, **p < 0.05, *p < 0.1.

**Table 2:** Factor loadings, convergent validity and discriminant validity results.

Scales	Indicators	S/L	SSL	SSSL	No. I	CR	AVE/CV	Sqrt AVE/DV	VIF
1. Technostress overload	TO P1	0.75	0.56	1.83	3	0.82	0.61	0.78	2.2
	TO P2	0.81	0.66						2.3
	TO P3	0.78	0.61						2.3
2. Technostress invasion	TI P1	0.89	0.80	1.52	2	0.86	0.76	0.87	2.9
	TI P2	0.85	0.72						2.9
3. Technostress complexity	TC P1	0.81	0.65	1.89	3	0.84	0.63	0.79	2.5
	TC P2	0.81	0.66						2.3
	TC P3	0.76	0.58						2.3
4. Technostress insecurity	TIS P1	0.74	0.55	1.85	3	0.83	0.62	0.79	2.2
	TIS P2	0.82	0.67						2.6
	TIS P3	0.79	0.62						2.4
5. Technostress uncertainty	TU P1	0.88	0.77	1.50	2	0.86	0.75	0.87	2.7
	TU P2	0.85	0.73						2.7
3. Psychological safety	PS 1	0.59	0.34	2.16	6	0.76	0.36	0.60	1.8
	PS 2	0.70	0.49						1.9
	PS 3	0.49	0.24						1.6
	PS 4	0.28	0.08						1.3
	PS 5	0.57	0.33						1.9
	PS 6	0.83	0.68						2.3
4. Intrinsic motivation	INT 1	0.79	0.62	2.39	3	0.92	0.80	0.89	2.8
	INT 2	0.95	0.90						5.9
	INT 3	0.93	0.87						5.7
5. Affective commitment	AFF 1	0.79	0.62	3.11	4	0.93	0.78	0.88	2.8
	AFF 2	0.88	0.77						4.1
	AFF 3	0.94	0.89						5.8
	AFF 4	0.91	0.83						5.1
6. Promotive voice	PMV 1	0.84	0.71	2.84	4	0.93	0.71	0.84	3.2
	PMV 2	0.89	0.79						3.8
	PMV 3	0.87	0.76						3.6
	PMV 4	0.76	0.58						2.4
7. Prohibitive voice	PHV 1	0.83	0.69	2.61	4	0.88	0.65	0.81	2.9
	PHV 2	0.89	0.78						3.5
	PHV 3	0.72	0.52						2.5
	PHV 4	0.79	0.62						2.8

S/L, standardized loadings; SSL, square standardized loadings; SSSL, sum of square standardized loadings; No. I., number of indicators; CR, composite reliability; AVE/CV, average variance extracted, Convergent Validity; SqrtAVE/DV, square root of average variance extracted, Discriminant Validity; VIF, variance inflation factor.

**Figure 1:**
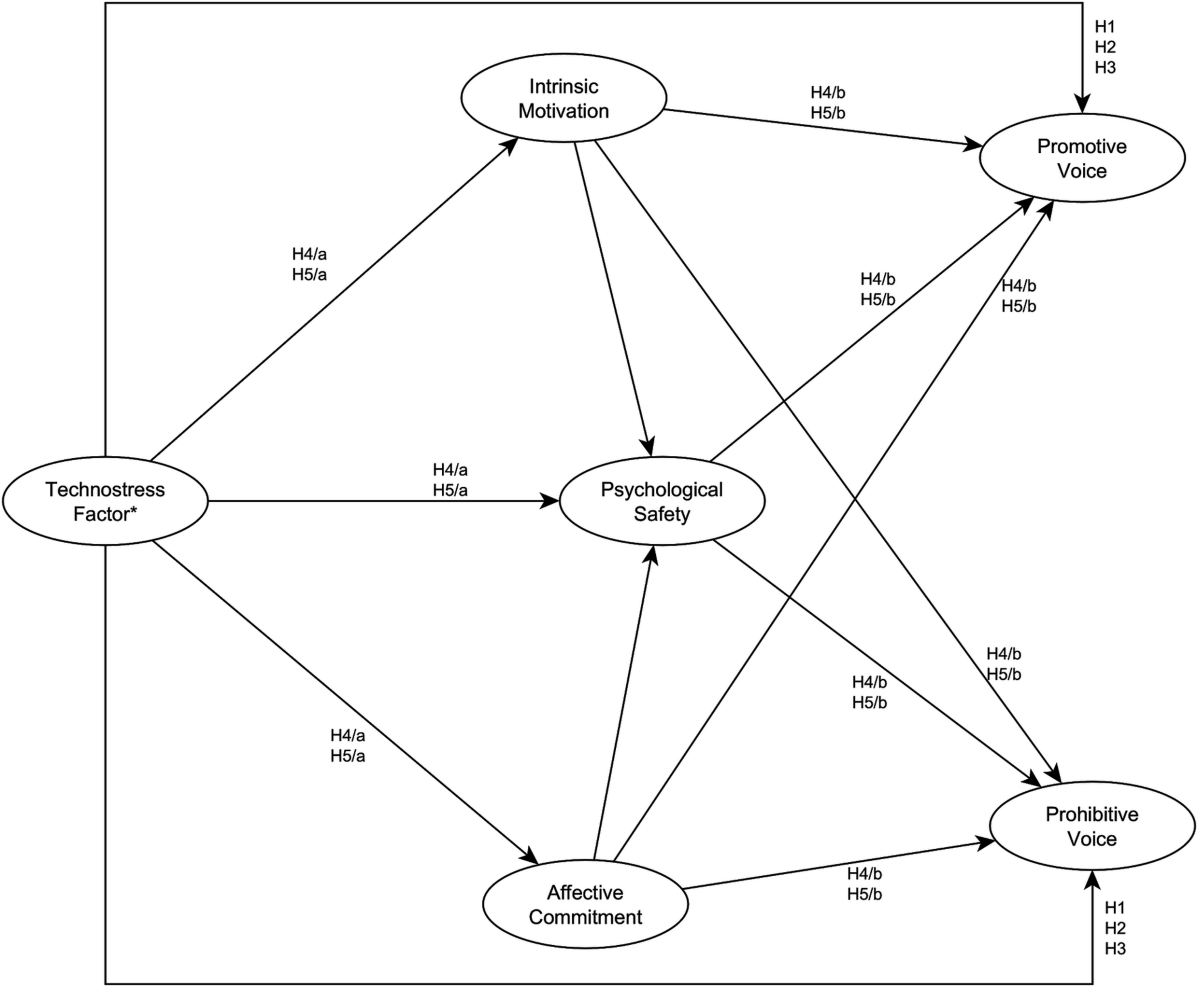
Schematic illustration of the indirect models. * We built five SEM models, each for every technostress factor. There were no other differences among the models. In these models, we focused on the indirect relationships between technostress and voice behavior.

To test the direct effects of technostress factors on voice behavior we conducted several three-step hierarchical regression models, the results of which are shown in Tables 3, 4. Then, we created five SEM models to test our indirect hypotheses according to Figure 1. These models, where we used psychological safety, intrinsic motivation, and affective commitment as moderators between technostress and voice behavior showed an acceptable fit to the data (see Table 5).

**Table 3:** Hierarchical regression model: variance explained in each step (promotive voice).

Promotive voice					
Regression steps	*R* ^2^	Δ *R*^2^	*F*	Δ *F*	Beta
Techno-overload					
Step 1 (control variables)	0.01		2.38		
Step 2 (linear effect of techno-overload)	0.02	0.00	1.89	0.90	0.05
Step 3 (quadratic effect of techno-overload)	0.02	0.00	1.68	1.06	0.05
Techno-invasion					
Step 1 (control variables)	0.01		2.38		
Step 2 (linear effect of techno-invasion)	0.01	0.00	1.71	0.38	0.03
Step 3 (quadratic effect of techno-invasion)	0.02	0.00	1.46	0.72	0.04
Techno-complexity					
Step 1 (control variables)	0.01		2.38		
Step 2 (linear effect of techno-complexity)	0.04	0.02	4.50**	8.63**	−0.17**
Step 3 (quadratic effect of techno-complexity)	0.04	0.00	3.49**	0.49	−0.04
Techno-insecurity					
Step 1 (control variables)	0.01		2.38		
Step 2 (linear effect of techno-insecurity)	0.05	0.04	6.28**	13.90**	−0.19**
Step 3 (quadratic effect of techno-insecurity)	0.07	0.02	6.61**	7.25**	0.19**
Techno-uncertainty					
Step 1 (control variables)	0.01		2.38		
Step 2 (linear effect of techno-uncertainty)	0.03	0.02	3.45*	5.51*	0.12*
Step 3 (quadratic effect of techno-uncertainty)	0.03	0.00	2.59*	0.06	−0.01

*p < 0.05, **p < 0.01; *R*^2^, explained variance of the dependent variable; Δ*R*^2^, the change of the explained variance between steps; Promotive voice is the dependent variable in all models shown in the table. Control variables were age and gender. In the third step, when we were interested in the quadratic effect, we added the squared term of the respective technostress factor to the model.

**Table 4:** Hierarchical regression model: variance explained in each step (prohibitive voice).

Prohibitive voice					
Regression steps	*R* ^2^	Δ*R*^2^	*F*	Δ*F*	Beta
Techno-overload					
Step 1 (control variables)	0.02		3.88*		
Step 2 (linear effect of techno-overload)	0.03	0.01	3.39*	2.37	0.09
Step 3 (quadratic effect of techno-overload)	0.03	0.00	2.69*	0.60	0.04
Techno-invasion					
Step 1 (control variables)	0.02		3.88*		
Step 2 (linear effect of techno-invasion)	0.02	0.00	2.74*	0.47	0.04
Step 3 (quadratic effect of techno-invasion)	0.02	0.00	2.23	0.69	0.04
Techno-complexity					
Step 1 (control variables)	0.02		3.88*		
Step 2 (linear effect of techno-complexity)	0.03	0.01	3.21*	1.85	−0.07
Step 3 (quadratic effect of techno-complexity)	0.03	0.00	2.48*	0.29	0.03
Techno-insecurity					
Step 1 (control variables)	0.02		3.88*		
Step 2 (linear effect of techno-insecurity)	0.02	0.00	2.87*	2.87*	−0.05
Step 3 (quadratic effect of techno-insecurity)	0.03	0.01	2.60*	1.72	0.10
Techno-uncertainty					
Step 1 (control variables)	0.02		3.88*		
Step 2 (linear effect of techno-uncertainty)	0.04	0.01	4.39**	5.31*	0.12*
Step 3 (quadratic effect of techno-uncertainty)	0.04	0.00	3.30*	0.07*	−0.01

*p < 0.05, **p < 0.01; *R*^2^, explained variance of the dependent variable; Δ*R*^2^, the change of the explained variance between steps; Prohibitive voice is the dependent variable in all models shown in the table. Control variables were age and gender. In the third step, when we were interested in the quadratic effect, we added the squared term of the respective technostress factor to the model.

**Table 5:** Summary of the model fit indices and explained variances.

Model	*χ2*	Df	*p*	CFI	TLI	RMSEA	Variance explained of PMV	Variance explained of PHV
Model-overload	621.897	278	<0.001	0.938	0.928	0.059	22.6%	19.5%
Model-invasion	547.662	254	<0.001	0.946	0.936	0.057	22.6%	19.8%
Model-complexity	632.647	278	<0.001	0.937	0.926	0.060	23.5%	20.5%
Model-insecurity	616.411	278	<0.001	0.939	0.929	0.058	23.3%	19.8%
Model-uncertainty	554.985	254	<0.001	0.944	0.934	0.057	22.8%	20.2%

χ2, chi-square; Df, degree of freedom; p, significance; CFI, comparative fit index; TLI, Tucker-Lewis index; RMSEA, root’/mean square error of approximation; PMV, promotive voice behavior; PHV, prohibitive voice behavior.

### Hypothesis testing

First, we examined the direct linear relationships between the factors of technostress and voice behavior (Tables 3, 4). The control variables together (age and gender) explained the 1.3% of the variance of promotive voice behavior and 2.1% of the variance of prohibitive voice. Then we entered the technostress factors (individually) into the regression equations.

In the case of challenge stressors, we observed a significant change in the explained variance (*Δ* R^2^) of promotive voice (2% - techno-uncertainty) and prohibitive voice (1% - techno-uncertainty) in addition to the variance explained by the control variables. Techno-uncertainty had a positive effect on promotive voice (*β* = 0.12 [95% CI: 0.01, 0.21], *p* = 0.022) and prohibitive voice (*β* = 0.12 [95% CI: 0.01, 0.23], *p* = 0.038), too. Based on these results, the first hypothesis (H1) was partially supported, as techno-uncertainty can positively predict both voice factors, but techno-overload did not have a significant direct effect on voice behavior.

As for hindrance stressors, we observed a significant change in the explained variance (Δ R^2^) of promotive voice (2% - techno-complexity; 4% - techno-insecurity) in addition to the variance explained by the control variables. In the variance of prohibitive voice, we did not observe any change. We found that techno-complexity (*β* = −0.16 [95% CI: −0.29, −0.05], *p* = 0.006) and techno-insecurity (*β* = −0.19 [95% CI: −0.36, −0.11], *p* < 0.001) influenced promotive voice negatively.

Therefore, our second hypothesis (H2) was also partially supported, as techno-complexity and techno-insecurity negatively predict promotive voice behavior. However, no other direct linear negative effects were observed.

**Figure 2:**
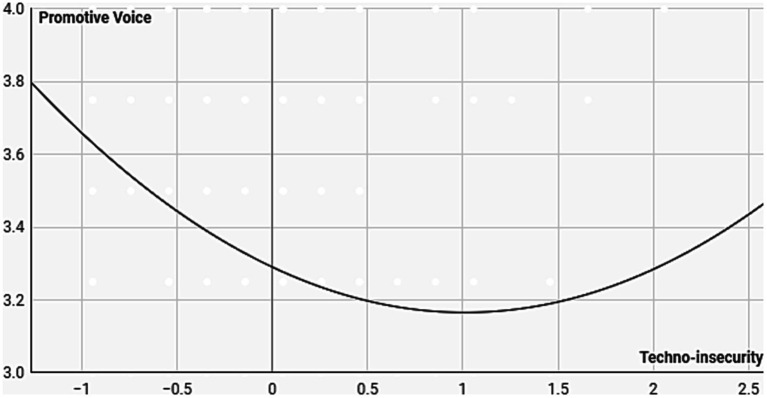
Curvilinear relationship between Techno-insecurity and Promotive Voice. The values on *x*-axis represent the mean-centered values of techno-insecurity. The original value corresponding to the mean-centered value of zero on *x*-axis is 1.94.

To assess the curvilinear relationships, we entered the squared term for each factor of technostress into the regression models, we used to examine H1 and H2. Among the hindrance stressors, for the squared term techno-insecurity, we observed a significant change in the explained variance (Δ *R*^2^) of promotive voice (2%), in addition to the variance explained by the control variables and techno-insecurity. In accordance with our expectation, we found that techno-insecurity (*β* = 0.19 [95% CI: 0.05, 0.33], *p* = 0.007) had a curvilinear relationship with promotive voice (Figure 2). The squared terms of the other hindrance stressors did not explain more variance of prohibitive voice than the control variables and the simple terms. All in all, H3 was partially supported, but only one factor, techno-insecurity had a weak curvilinear relationship with promotive voice.

We also examined the curvilinear effects of challenge stressors. In their case, when we investigated the effect of the squared terms, we did not find any significant change in the explained variance (Δ R^2^) of promotive voice and prohibitive voice in addition to the variance explained by the control variables and techno-overload and techno-uncertainty. The results are summarized in Tables 3, 4.

Then we observed the indirect effects of the technostress factors in five separate SEM models. The moderator variables (intrinsic motivation, affective commitment, psychological safety) and the outcome variables (promotive voice behavior, prohibitive voice behavior) were the same in all models. To test H4/a, and H4/b, we used the first and the fifth models (Model-Overload and Model-Uncertainty, see Table 5.). According to these models, techno-overload positively predicts intrinsic motivation (*β* = 0.16 [95% CI: 0.03, 0.27], *p* = 0.013) and negatively predicts psychological safety (*β* = −0.29 [95% CI: −0.41, −0.17], *p* < 0.001), but it did not have a significant effect on affective commitment (*β* = 0.06 [95% CI: −0.05, 0.17], *p* = 0.308). On the other hand, techno-uncertainty predicted positively both intrinsic motivation (*β* = 0.11 [95% CI: 0.04, 0.27], *p* = 0.001) and affective commitment (*β* = 0.10 [95% CI: 0.04, 0.22], *p* = 0.011), but it did not have a significant effect on psychological safety (*β* = 0.06 [95% CI: −0.03, 0.17], *p* = 0.151) Therefore, H4/a was partially confirmed. Then, we investigated the two models together to test H4/b. Through intrinsic motivation, both techno-overload and techno-uncertainty had an indirect positive effect on promotive voice behavior (techno-overload - intrinsic motivation—promotive voice: *β* = 0.04 [95% CI: 0.01, 0.09], *p* = 0.006; techno-uncertainty - intrinsic motivation—promotive voice: *β* = 0.03 [95% CI: 0.01, 0.08], *p* = 0.017). Moreover, techno-uncertainty had a positive effect through affective commitment as well, on promotive-and prohibitive voice (techno-uncertainty—affective commitment—psychological safety—promotive voice: *β* = 0.04 [95% CI: 0.01, 0.04], *p* = 0.006; techno-uncertainty—affective commitment—psychological safety—prohibitive voice: *β* = 0.04 [95% CI: 0.01, 0.05], *p* = 0.008). Therefore, H4/b was also partially supported. However, in the case of techno-overload, which did not have a significant direct effect on voice, we observed a negative impact on psychological safety which mediated, hence it had an indirect negative effect on both voice factors (techno-overload - psychological safety - promotive voice: *β* = −0.0930 [95% CI: −0.154, −0.0544], *p* < 0.001; techno-overload - psychological safety—prohibitive voice: *β* = −0.1036 [95% CI: −0.20, −0.0851], *p* < 0.001).

To test H5/a, and H5/b we used the second, third, and fourth models (Model-Invasion, Model-Complexity, and Model-Insecurity, see Table 5.) All three factors had a significant negative effect on psychological safety (techno-invasion—psychological safety: *β* = −0.27 [95% CI: −0.38, −0.14], *p* < 0.001; techno-complexity—psychological safety: *β* = −0.23 [95% CI: −0.35, − < 0.001]; techno-insecurity—psychological safety: *β* = −0.32 [95% CI: −0.43, −0.21], *p* < 0.001). Intrinsic motivation and affective commitment were only influenced significantly and negatively by techno-insecurity (techno-insecurity—intrinsic motivation: *β* = −0.17 [95% CI: −0.29, −0.04], *p* = 0.014; techno-insecurity—affective commitment: *β* = −0.16 [95% CI: −0.28, −0.03], *p* = 0.015). Thus, H5/a was also partially supported.

Then, to test H5/b, we investigated the indirect effects in the three models. Through psychological safety, all the hindrance stressors had significant indirect negative effect on promotive voice (techno-invasion—psychological safety—promotive voice: *β* = −0.08 [95% CI: −0.13, −0.04], *p* < 0.001; techno-complexity—psychological safety—promotive voice: *β* = −0.07 [95% CI: −0.13, −0.03], *p* < 0.001; techno-insecurity—psychological safety—promotive voice: *β* = −0.10 [95% CI: −0.17, −0.06], *p* < 0.001), and on prohibitive voice, too (techno-invasion—psychological safety—prohibitive voice: *β* = −0.10 [95% CI: −0.16, −0.06], *p* < 0.001; techno-complexity—psychological safety—prohibitive voice: *β* = −0.08 [95% CI: −0.17, −0.05], *p* < 0.001; techno-insecurity—psychological safety—prohibitive voice: *β* = −0.10 [95% CI: −0.22, −0.08], *p* < 0.001). In the case of techno-insecurity, intrinsic motivation also moderated the negative effect on promotive voice (techno-insecurity—intrinsic motivation—promotive voice: *β* = −0.04 [95% CI: −0.09, −0.01], *p* = 0.010). Therefore, H5/b was also partially supported. Table 6 summarizes the examination of all our hypotheses.

**Table 6:** Hypotheses summary.

Abb.	Hypotheses	*β-value*	95% CI	*p*-value	Decision
Linear direct effects					
H1	T. overload ➔ PMV	0.05	−0.05, 0.15	0.329	Partially supported
	T. overload ➔ PHV	0.08	−0.03, 0.20	0.134	
	T. uncertainty ➔ PMV*	0.12	0.01, 0.21	0.022	
	T. uncertainty ➔PHV*	0.12	0.01, 0.23	0.038	
H2	T. invasion ➔ PMV	0.03	−0.07, 0.13	0.552	Partially supported
	T. invasion ➔ PHV	0.04	0.07, 0.14	0.489	
	T. complexity ➔ PMV*	−0.16	−0.29, −0.05	0.006	
	T. complexity ➔ PHV	−0.07	−0.21, 0.05	0.207	
	T. insecurity ➔ PMV*	−0.19	−0.36, −0.11	<0.001	
	T. insecurity➔ PHV	−0.05	−0.20, 0.08	0.385	
H4/a	T. overload ➔ INT*	0.16	0.03, 0.27	0.013	Partially supported
	T. overload ➔ AFF	0.06	−0.05, 0.17	0.308	
	T. overload ➔ PS*	−0.29	−0.41, −0.17	<0.001	
	T. uncertainty ➔ INT*	0.11	0.04, 0.27	0.001	
	T. uncertainty ➔ AFF*	0.10	0.04, 0.22	0.011	
	T. uncertainty ➔ PS	0.06	−0.03, 0.17	0.151	
H5/a	T. invasion ➔ INT	0.09	−0.04, 0.23	0.165	Partially supported
	T. invasion ➔ AFF	0.09	−0.03, 0.03	0.138	
	T. invasion ➔ PS*	−0.27	−0.38, −0.14	<0.001	
	T. complexity ➔ INT	−0.06	−0.18, 0.07	0.355	
	T. complexity ➔ AFF	−0.05	−0.17, 0.18	0.435	
	T. complexity ➔ PS*	−0.23	−0.35, −0.10	0.001	
	T. insecurity ➔ INT*	−0.17	−0.29, −0.04	0.014	
	T. insecurity ➔ AFF*	−0.16	−0.28, −0.03	0.015	
	T. insecurity ➔ PS*	−0.32	−0.43, −0.21	<0.001	
Curvilinear direct effects					
H3	T. invasion ➔ PMV	0.04	−0.06, 0.14	0.396	Partially supported	T. invasion ➔ PHV	0.04	−0.06, 0.15	0.406	T. complexity ➔ PMV	−0.04	−0.15, 0.07	0.485	T. complexity ➔ PHV	0.03	−0.09, 0.15	0.589	T. insecurity ➔ PMV*	0.19	0.05, 0.33	0.007	T. insecurity ➔ PHV	0.10	−0.05, 0.25	0.190
	Linear indirect effects					
	H4/b	T. overload ➔ INT ➔ PMV*	0.04	0.01, 0.09		0.006	Partially supported
		T. overload ➔ INT ➔ PHV	0.02	0.00, 0.07		0.061	
		T. overload ➔ AFF ➔ PMV	0.00	−0.01, 0.02		0.580	
		T. overload ➔ AFF ➔ PHV	0.00	−0.02, 0.01		0.871	
T. overload ➔ PS ➔PMV*		−0.09	−0.15, −0.05	<0.001			
T. overload ➔ PS ➔ PHV*		−0.10	−0.20, −0.08	<0.001			
T. uncertainty ➔ INT ➔ PMV*		0.03	0.01, 0.08	0.017			
T. uncertainty ➔ INT ➔ PHV		0.01	−0.00, 0.05	0.155			
T. uncertainty ➔ AFF ➔ PMV		0.00	−0.02, 0.03	0.746			
T. uncertainty ➔ AFF ➔ PMV		−0.00	−0.02, 0.03	0.901			
T. uncertainty ➔ PS ➔ PMV		0.02	0.00, 0.07	0.101			
T. uncertainty ➔ PS ➔ PMV		0.02	−0.00, 0.09	0.115			
H5/b	T. invasion ➔ INT ➔ PMV	0.03	0.00, 0.06	0.099	Partially supported
	T. invasion ➔ INT ➔ PHV	0.01	0.00, 0.05	0.120	
	T. invasion ➔ AFF ➔ PMV	0.00	−0.00, 0.02	0.598	
	T. invasion ➔ AFF ➔ PHV	0.00	−0.01, 0.01	0.868	
	T. invasion ➔ PS ➔PMV*	−0.08	−0.13, −0.04	<0.001	
	T. invasion ➔ PS ➔ PHV*	−0.10	−0.16, −0.06	<0.001	
	T. complexity ➔ INT ➔ PMV	−0.02	−0.05, 0.01	0.316	
	T. complexity ➔ INT ➔ PHV	−0.01	−0.04, 0.00	0.282	
	T. complexity ➔ AFF ➔ PMV	−0.00	−0.02, 0.00	0.611	
	T. complexity ➔ AFF ➔ PHV	0.00	0.01, 0.02	0.759	
	T. complexity ➔ PS ➔PMV*	−0.07	−0.13, −0.03	<0.001	
	T. complexity ➔ PS ➔ PHV*	−0.08	−0.17, −0.05	<0.001	
	T. insecurity ➔ INT ➔ PMV	−0.04	−0.09, −0.01	0.010	
	T. insecurity ➔ INT ➔ PHV	−0.03	−0.07, −0.00	0.083	
	T. insecurity ➔ AFF ➔ PMV	−0.00	−0.04, 0.02	0.751	
	T. insecurity ➔ AFF ➔ PHV	0.00	−0.03, 0.03	0.930	
	T. insecurity ➔ PS ➔PMV	−0.10	−0.17, −0.06	<0.001	
	T. insecurity ➔ PS ➔ PHV	−0.10	−0.22, −0.08	<0.001	

Abb., hypothesis abbreviation; Hypotheses, hypotheses and sub-hypotheses; CI, confidence interval; *p*-value, significance level; PMV, Promotive Voice Behavior; PHV, Prohibitive Voice Behavior; INT, Intrinsic Work Motivation; AFF, Affective Organizational Commitment; PS, Psychological Safety; *: significant effect.

## Discussion

We conducted a study to examine how technostress affects organizational voice behavior. Drawing from the challenge-hindrance stressor framework (Cavanaugh et al., 2000), we hypothesized that technostress could have both challenge and hindrance effects. In accordance with the findings of Chandra et al. (2019) and considering the items from the Technostress Creators Inventory, we presumed that techno-overload and techno-uncertainty serve as challenge stressors, while techno-complexity, techno-invasion, and techno-insecurity act as hindrance stressors. Furthermore, we postulated that challenge stressors positively predict voice behavior, while hindrance stressors negatively predict it (Chandra et al., 2019; Zhou et al., 2019). Additionally, we explored whether hindrance stressors exhibit a U-shaped relationship with voice behavior (Zhou et al., 2019). Our analysis not only examined the direct impact of technostress on voice behavior but also assessed the indirect effects through three variables: intrinsic motivation, affective commitment, and psychological safety (Buzás and Faragó, 2023; Podsakoff et al., 2007).

Techno-uncertainty is the most prominent challenge stressor, it has a direct, positive effect on both promotive and prohibitive voice behavior, as well as on intrinsic motivation and affective commitment. This suggests that employees view innovation and technological development as opportunities for growth and advancement rather than threats to their psychological safety. They feel encouraged to express their ideas and criticisms in an environment that fosters continuous learning and improvement.

On the other hand, Techno-overload, which refers to the increased pace of work due to technology, can diminish psychological safety and inhibit voicing behaviors. However, it can also increase intrinsic motivation among employees, leading to a greater intention to contribute positively to the organization with their suggestions. Despite this, criticism may be less likely to be voiced under conditions of Techno-overload. The impact of these stressors depends on the organizational climate. If the organization promotes intrinsic motivation and maintains a positive psychological climate, Techno-overload may be perceived as a challenge stressor rather than a hindrance stressor. However, if psychological safety is compromised, it can become a hindrance stressor, impeding employee performance and well-being.

Techno-insecurity, which threatens an employee’s position and status in the workplace, is indeed a hindrance stressor. It, directly and indirectly, impedes promotive voice behavior by reducing both intrinsic motivation and psychological safety. Techno-complexity, which involves employees feeling insufficiently skilled or time-constrained to handle technological advancements, is indeed a hindrance stressor. It directly diminishes promotive voice behavior and negatively impacts prohibitive voice behavior through its influence on psychological safety. Techno-invasion is not a significant hindrance stressor. Its effect on voice behavior primarily occurs through its negative impact on psychological safety rather than directly impeding promotive or prohibitive voice behavior. Simon et al. (2024) found that techno-complexity and techno-insecurity have a significant indirect impact on the desire to work from home, which also underscores the hindering nature of these technostress factors.

For hindrance stressors, the relationship between techno-complexity, techno-insecurity, and techno-invasion on both promotive and prohibitive voice behavior was moderated by psychological safety. Additionally, intrinsic motivation moderated the relationship between techno-insecurity and promotive voice behavior. In this scenario, feelings of insecurity resulted in decreased intrinsic motivation, leading to fewer instances of expressing opinions These findings are consistent with previous research where intrinsic motivation also played a significant role in predicting voice (Buzás and Faragó, 2023; Jaaffar and Samy, 2023; Uğurlu and Ayas, 2016). Moreover, affective commitment primarily influenced psychological safety, aligning with the results of our previous research (Buzás and Faragó, 2023).

Our findings underscore the importance of psychological safety, which aligns with prior research (Buzás and Faragó, 2023; Detert and Burris, 2007; Liang et al., 2012; Walumbwa and Schaubroeck, 2009). We observed that all technostress factors had a negative predictive impact on psychological safety, except for techno-uncertainty.

We confirm the usefulness of distinguishing between the two forms of voice—promotive and prohibitive—in connection with the impact of technostress factors. In the presence of the unambiguous challenge stressor, techno uncertainty, both promotive and prohibitive voice are enhanced; however, the less clear challenge stressor, techno overload increases only the promotive voice while hindering the prohibitive voice. Hindrance stressors negatively influence only the promotive voice, meaning that in their presence, suggestions diminish while criticisms remain unchanged.

To the best of our knowledge, Chandra et al. (2019) is the only researcher who has investigated the challenge-hindrance effect of technostress on a non-efficiency-based organizational behavior—specifically, innovation. By comparing our results with those of Chandra et al. (2019), we aim to emphasize the challenge-hindrance nature of individual technostress factors and identify potential cultural differences in responding to technostress. Given that voice behavior, like innovation, pertains to non-efficiency-based behavior, we argue that this comparison is valid.

First, we found a difference in which of the five technostress factors were identified as challenge stressors and which as hindrance stressors. Regarding linear effects, techno-uncertainty can unequivocally be classified as a challenge stressor in both studies. The ambiguity of techno-overload is also evident in both studies: Chandra did not find any linear relationship between innovation and techno-overload, whereas we observed indirect effects on voice, moderated by intrinsic motivation and psychological safety. Techno-complexity consistently shows a negative linear connection in both samples, indicating cross-study consistency. Additionally, the negative effect of techno-invasion is more pronounced in Chandra’s study than in ours. The most significant difference between our results and those of Chandra et al. (2019) regarding hindrance stressors lies in techno-insecurity. In our study, it has a direct negative impact on promotive voice, intrinsic motivation, and psychological safety, firmly establishing it as a hindrance stressor. However, in Chandra’s study, it does not exhibit any significant effect, and thus, it does not qualify as a hindrance stressor. The items in the techno-insecurity subscale reflect fear of job loss due to an inability to adapt to recent technology. Interestingly, in Chandra’s research, techno-insecurity does not qualify as a hindrance stressor.

Second, we examined the differences in curvilinear relationships. While Chandra found a U-shaped relationship for all hindrance stressors, our study revealed a weak U-shaped relationship only between techno-insecurity and voice behavior.

To compare our results with Chandra’s findings, it is important to note that Chandra’s subjects were senior managers in Europe and Asia, whereas our study focused on a homogeneous Hungarian employee sample with diverse employment statuses. These differences may stem from the status differences between the subjects in the two studies. Chandra notes that senior-level managers, due to their status and experience, tend to perceive greater job security and are better equipped to cope proactively with stressful situations.

Broader cultural differences may also play a role. Voice behavior reflects individuals’ resilience in stressful situations: Do they believe they can alter challenging circumstances by proposing solutions, or do they highlight detrimental stress and its causes? The Conservation of Resources (COR) theory posits that individuals’ motivation to safeguard and augment their accrued resources aids them in coping with stress. This suggests that individuals are motivated to voice their concerns when stress levels are either low or high but refrain from doing so when stress is moderate. As techno-insecurity increases, subjects initially become reluctant to engage in voice behavior due to concerns about resource depletion (resource conservation). However, beyond a certain threshold, they once again engage in voice behavior to ensure future functionality (resource acquisition).

When evaluating the impact of cultural context on responses to adverse situations, it is noteworthy that Hungarians rank among the most pessimistic populations in Europe, as recurring Eurobarometer surveys indicate. Adapting to adverse situations often involves risk-taking. In Hungary—a country characterized by significant power distance—expressing concerns and suggestions to superiors entails greater risk compared to cultures with lower power distance. This cultural context influences individuals’ inclination toward risk-taking. A Hungarian field experiment found that risk-taking decreases as resources diminish, suggesting that individuals with scarce resources tend to avoid risk rather than seek solutions (Faragó and Uatkán, 2018). The significant impact of psychological safety on voice behavior and the scarcity of U-shaped relationships between hindrance stressors and voice behavior support this conclusion: individuals require a secure environment to feel safe taking the risk of voicing openly their opinions.

However, while our comparison with Chandra’s findings is informative and meaningful, it must be interpreted with caution. The comparison is not based on a well-planned experimental design; we analyzed data where the technostress questionnaire was the same, but the dependent variables were only similar in that they reflected non-efficiency-based OCB behavior. Additionally, the samples differed, with Chandra surveying managers and our study focusing on employees. Despite these differences, we believe this tentative comparison is valuable in highlighting the diversity of responses to technostress and the potential cultural determinants of adaptation to stressful situations.

## Conclusion

### Theoretical implications

When employees have adequate control and resources to cope with stressful situations, they tend to perceive these situations as positive challenges, making them more likely to engage in Organizational Citizenship Behavior (OCB), such as voice behavior. Conversely, when they perceive a lack of control and limited resources, they are less likely to engage in costly voice behavior. Karasek (1979) Demand-Control model suggests that individuals cope with stressors by incorporating their capabilities and seeking autonomy and control. For challenging stressors, individuals perceive work demands as controllable, resulting in a positive relationship with performance. In contrast, hindrance stressors lead to reduced efforts and a negative relationship with performance (Edwards et al., 2014; Theorell et al., 1990; Wallace et al., 2009). The Demand-Control model predicts a linear relationship between technostress and OCB behavior.

Based on the control theory (Karasek, 1979), we can infer whether each technostress factor functions as a hindrance or challenge stressor. Our findings confirm our expectation that technostress comprises both challenge and hindrance factors. Techno-overload and techno-uncertainty are perceived as positive challenges, increasing intrinsic motivation, and encouraging greater effort, including voice behavior. In contrast, hindrance stressors like techno-complexity and techno-insecurity are perceived as uncontrollable threats, reducing motivation to overcome them. Promotive voice is more strongly affected by technostress than prohibitive voice, showing both positive and negative influences.

Examining curvilinear relationships between technostress and voice behavior provided additional insights. According to COR theory, individuals’ responses to stress are dynamic—they strive to preserve and protect resources while also seeking to accumulate more. This perspective helps explain how individuals cope with stress and how cultural factors shape coping strategies. We propose that status and cultural influences play a role in shaping individuals’ reactions to challenge and hindrance stressors. The U-shaped relationship between hindrance stressors and positive organizational behavior suggests proactive, resilient adaptation to environmental stress. However, among Hungarian participants this U-shaped relationship was weak, indicating lower resilience and a limited ability to cope with stress. A broader societal example illustrates this pattern: despite Hungary’s political and economic challenges (e.g., the highest inflation rate in the EU, corruption, authoritarian decision-making, and a lack of dialogue with civil society), there has been insufficient solidarity, voicing, and protest.

### Practical implications

Several practical recommendations emerge from our findings. It is crucial for employees in technology-intensive roles to perceive IS use as a challenge rather than a hindrance (Schoch, 2023). Organizations should provide training to facilitate the smooth adoption of recent technologies, minimize strain, and reinforce psychological safety (White and Bruton, 2010). Employees should be prepared for the temporary efficiency loss associated with technological changes. Additionally, self-efficacy development programs and intrinsic motivation strategies can help mitigate technostress. Encouraging voice behavior can also transform technostress into an opportunity for organizational improvement.

### Limitations

First, our research was based on a cross-sectional sample, preventing us from establishing causal relationships. Some authors dispute the applicability of cross-sectional designs (Law et al., 2016). Therefore, it would be important to examine the questions we raised using a longitudinal design or in an experimental setting, as well. Second, we conducted questionnaire-based data collection, which might have introduced biases (e.g., social desirability). Third, despite our efforts to include employees from various fields, our sample was not fully representative. Future research should replicate our study with a larger sample size and induce participants from different nationalities. Fourth, there is currently no validated Hungarian version of the Technostress Creators Inventory, the Voice Scale, or the items related to psychological safety, although the translation process followed the principles outlined by Beaton et al. (2000). Fifth, we did not measure IS use appraisal and the challenge and hindrance appraisals which could provide further insights into the nature of technostress within the challenge-hindrance dimension. Asking direct questions such as, “Does IS help me to voice my opinion?” might have led respondents to focus solely on the narrow technical opportunities provided by the technology, rather than on their general willingness to voice their opinions. Additionally, did not assess the technological development level of the organizations where the participants work. Sixth, voice behavior may not only appear as an outcome variable in relation to stress. It is conceivable that employees use voice behavior as a means to alleviate tension. Furthermore, voice may lead to solutions that ultimately reduce technostress.

Finally, the chosen analytical approach warrants discussion. We employed moderation models to examine the mechanism underlying the relationship between technostress and voice behavior. However, this method is debated in methodological literature. Some scholars claim it to be unreliable (Bullock et al., 2010; Bullock and Green, 2021), and highlight its analytical limitations (Imai et al., 2010). Despite these criticisms moderation and mediation models are still widely used in social studies due to their simplicity (Wittmann and Wulf, 2023; Zhou et al., 2023). Hayes (2022) argues that implicit mediation procedures are among the best and most widely used choices in the social sciences. He further suggests that mediation models offer greater reliability when indirect effects are thoroughly explored. Nonetheless, there are known methods to enhance the reliability of these models. Gerber and Green (2012) propose implicit mediation analysis, where the effect of the input variable is analyzed in more detail and tested on separate subsamples. If the magnitude of the independent variable’s effect changes, the effect on the mediator is expected to adjust accordingly. Due to our low sample size, we were unable to analyze our data according to Gerber and Green’s approach, as creating subsamples would have resulted in excessively small groups. However, we consider it important for future research to investigate this issue using the suggested analyses.

### Future research directions

Emotions should not be disregarded in the examination of the relationship between technostress and voice behavior. Spector and Goh (2001) delve into the role of emotion in the occupational stress process arguing that job stressors provoke behavioral strain responses. Emotions, as motivational drivers of purposeful action, play a pivotal role in the stress-behavioral response link. Moreover, the cognitive interpretation of the situation and the motivation for action are crucial determinants of resulting emotions. While stressors are subjective, it is meaningful to consider factors that are perceived as environmental or objective job stressors. This perspective supports further differentiation of challenge and hindrance stressors in the occupational stress field.

Future studies should explore the cognitive and emotional aspects of informational system use. Longitudinal research could offer valuable insights into this area as well. Additionally, as we tentatively suggested, cultural differences may influence problem-focused coping strategies for occupational technostress Therefore, it would be valuable to investigate the impact of technostress on voice behavior in different cultural contexts.

## Data Availability

The datasets presented in this study can be found in online repositories. The names of the repository/repositories and accession number(s) can be found in the article/Supplementary material.
