# Supplementary material for: Navigating the digital landscape: unraveling the interplay of challenge and hindrance components of technostress on employee voice behavior
Source: Front Psychol. 2025 Mar 26;16:1434275. doi: 10.3389/fpsyg.2025.1434275 (PMC11980636; doi:10.3389/fpsyg.2025.1434275)
Supplement: Supplementary file 1 [file Table_1.DOCX]

# Appendices

## Data Transparency Table

| Variables in the Complete Dataset | MS 1 (Simon et al.)  (STATUS= in review process) | MS 2  (STATUS = current) |
| --- | --- | --- |
| Sex | x | x |
| Age | x | x |
| Occupation | x | x |
| Position | x | x |
| Region |  |  |
| Sector | x | x |
| Education | x | x |
| Marital status |  |  |
| Organizational culture (1-4)* |  |  |
| Leader openness |  |  |
| Leader technical openness |  |  |
| Home office related questions (1-5)* | x | x |
| Latent and Manifest Benefits Scale (1-19)* | x |  |
| Technostress Creators Inventory (1-23)* | x | x |
| Technostress Inhibitors (1-13)* |  |  |
| Employee Voice Behavior Scale (1-8)* |  | x |
| Psychological Safety (1-6)* |  | x |
| Multidimensional Work Motivation Scale (1-19) |  | x |
| Three-Component Model Employee Commitment Scale (1-12) |  | x |

*Note:* * number of questions/items of an instrument. Data created: 2022.
